# Protein crystallography beamline BL2S1 at the Aichi synchrotron

**DOI:** 10.1107/S1600577516018579

**Published:** 2017-01-01

**Authors:** Nobuhisa Watanabe, Takayuki Nagae, Yusuke Yamada, Ayana Tomita, Naohiro Matsugaki, Masao Tabuchi

**Affiliations:** a Synchrotron Radiation Research Center, Nagoya University, Nagoya 464-8603, Japan; b Department of Biotechnology and Biomaterial Chemistry, Graduate School of Engineering, Nagoya University, Nagoya 464-8603, Japan; c Photon Factory, Institute of Materials Structure Science, High Energy Accelerator Research Organization, Tsukuba, Ibaraki 305-0801, Japan

**Keywords:** protein crystallography, superbend beamline, high pressure

## Abstract

The protein crystallography beamline BL2S1, constructed at one of the 5 T superconducting bending-magnet ports of the Aichi synchrotron, is available to users associated with academic and industrial organizations. The beamline is mainly intended for use in X-ray diffraction measurements of single-crystals of macromolecules such as proteins and nucleic acids. Diffraction measurements for crystals of other materials are also possible, such as inorganic and organic compounds. BL2S1 covers the energy range 7–17 keV (1.8–0.7 Å) with an asymmetric-cut curved single-crystal monochromator [Ge(111) or Ge(220)], and a platinum-coated Si mirror is used for vertical focusing and as a higher-order cutoff filter. The beamline is equipped with a single-axis goniometer, a CCD detector, and an open-flow cryogenic sample cooler. High-pressure protein crystallography with a diamond anvil cell can also be performed using this beamline.

## Abstract 2

The protein crystallography beamline BL2S1 has been constructed on the 5 T superbend port of the AichiSR.

## 1. Introduction   

The Aichi synchrotron (AichiSR) in Seto City, Aichi Prefecture, Japan, is a small electron storage ring designed for 1.2 GeV beam energies with a natural beam emittance of 53 nm rad and 1% coupling. The Synchrotron Radiation Research Center, Nagoya University, has fully supported the facility since its planning phase. The facility comprises a 50 MeV linac, a 1.2 GeV booster synchrotron and a 1.2 GeV storage ring of 72 m circumference, and is operated in a 300 mA top-up injection mode (Yamamoto *et al.*, 2010 ▸). Initially, six beamlines were constructed and have been in service since March 2013 (Tabuchi *et al.*, 2016 ▸). The ring has 12 bending magnets, of which eight are normal bending magnets of 1.4 T and four are superconducting bending magnets of 5 T. The 5 T superbends provide X-rays with a critical energy of 4.8 keV (Yamamoto *et al.*, 2011 ▸). The facility is owned by the Aichi Science and Technology Foundation, which cooperates with industries, academic organizations and the Aichi Prefectural Government. Normal user-operation of the facility lasts 8.5 h a day, from 10:00 am to 6:30 pm, and four days a week (Tuesday to Friday). On Mondays, the accelerator group generally conduct machine study experiments. A new beamline BL2S1 was recently designed for use in macromolecular crystallography, and has been constructed and is operated by the Nagoya University. The beamline has been open for general users since May 2015.

## 2. Beamline overview   

### 2.1. Beamline optics   

**Figure 1:**
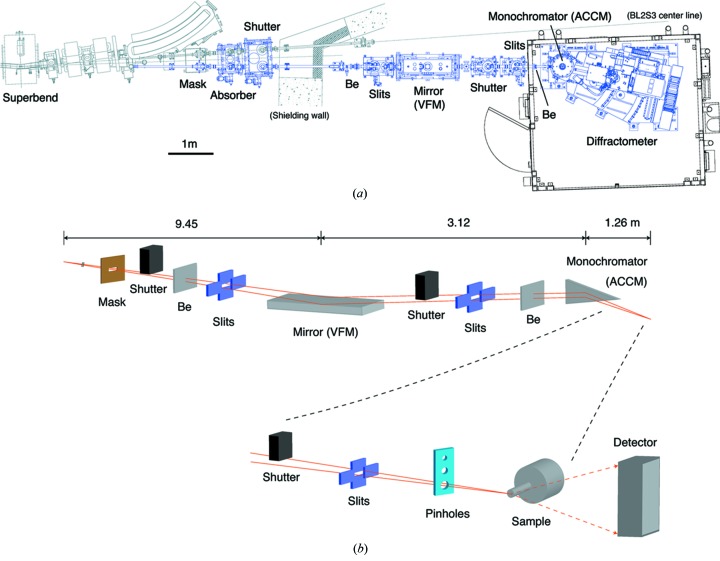
Layout of BL2S1. (*a*) Drawing of the beamline, and (*b*) schematic of the layout of the various optical components.

The photon source of BL2S1 is the 5 T superconducting bending magnet. The synchrotron radiation from the source (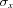, 0.33 mm; 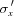, 0.18 mrad; 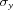, 0.046 mm; 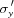, 0.011 mrad; *x*, horizontal; *y*, vertical) is horizontally divided at the beamline front-end, where 2.0 mrad is provided to the 2S1 branch. The first 200 µm-thick beryllium window separates the storage ring vacuum from that of the beamline and also acts as a high-pass filter. The layouts of the beamline components and the optical alignment of BL2S1 are shown schematically in Fig. 1 ▸, and the beamline parameters are summarized in Table 1 ▸. The beamline optics comprise a vertical-focusing bent-plane mirror (VFM, water-cooled) and an asymmetric-cut curved single-crystal monochromator (ACCM, no cooling) for monochromatization and horizontal focusing of the beam. Historically, many protein crystallography beamlines have used this type of monochromator to achieve a focused monochromatic beam (Lemonnier *et al.*, 1978 ▸). Using a single-crystal monochromator, the direction of the beam is changed with wavelength. However, this type of beamline design enables construction of several branch beamlines on a single bending-magnet section of a smaller ring. It has therefore been popular in second-generation synchrotron facilities (Helliwell, 2006 ▸), which are relatively compact compared with the current third-generation low-emittance storage rings. In Japan, BL2S1 resembles the former BL6A at the Photon Factory (Satow *et al.*, 1989 ▸).

The VFM has a platinum-coated 1 m-long reflecting surface and is installed at 9.45 m from the source, at about 2:1 focusing position. The VFM also works as a low-pass filter. With a glancing angle of 4.0 mrad, it reduces flux at a spectral range higher than 21 keV, which could have passed through the single-crystal monochromator as higher harmonics.

**Figure 2:**
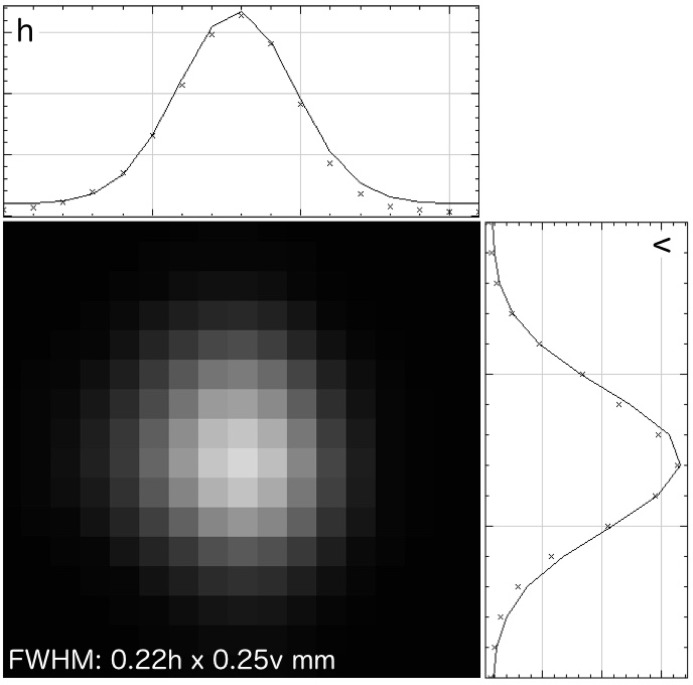
Typical focusing profile of the 1.12 Å beam at the sample position without the pinhole collimator. This image was obtained using the Remote RadEye1 detector (Teledyne DALSA, Canada). The pixel size of the detector is 48 µm.

The ACCM is installed inside the experimental hutch at 12.57 m from the source, and the distance from the ACCM to the sample position is 1.26 m. Another 200 µm-thick beryllium window separates the high vacuum of the beamline from the ACCM chamber filled with helium gas at atmospheric pressure. In order to use the central part of the cylindrically bent 20 cm-long ACCM crystal, the horizontal beam divergence is reduced to 1.2 mrad by the slit before the VFM. Several triangular Ge(111) and (220) crystals with different asymmetric angles are used to achieve optimum 10:1 focusing of the horizontally spread synchrotron X-ray beam of the storage ring of AichiSR onto the sample position without increasing the beam divergence. The existing monochromator crystals and their asymmetric angles are listed in Table 2 ▸. In order to achieve a beam demagnification ratio of 10:1 at the designed wavelength, each crystal has to be cut with a specific asymmetric angle. Currently, the standard monochromator crystal of BL2S1 is Ge(111) with an asymmetric angle of 7.61°, because most users of protein crystallography use X-rays near 1.0 Å (12.4 keV). Although the optimum wavelength of the crystal is 1.05 Å (11.8 keV), the crystal is used at a wavelength of 1.12 Å (11.07 keV), just below the *K*-absorption edge, in order to avoid X-ray absorption of germanium. Its distinctive focusing profile, where the beam is compressed horizontally to the same size as the vertical extent, is shown in Fig. 2 ▸. The focusing profiles of other monochromator crystals are also similar if we use the ACCM near its theoretical optimum wavelength. It is necessary to exchange the monochromator crystals by hand when changing the X-ray wavelength by a large amount; this is a half-day job for facility staff including optical alignments. We do not use the rotated-inclined focusing monochromator technique that enables simultaneous tuning of the asymmetry factor and the radius of curvature of the crystal over a wide wavelength range (Watanabe *et al.*, 1999 ▸), because obtaining a large enough germanium crystal is impractical. Therefore, it is necessary to make a monochromator crystal that has a specific asymmetric angle if users want to use X-rays at some absorption edge with a fine energy resolution. However, we think that this is rarely requested nowadays because the single-wavelength anomalous diffraction (SAD) method using X-ray wavelengths far from absorption edges is gradually becoming more popular than the multi-wavelength anomalous diffraction (MAD) method.

The VFM bender and other beamline components, such as beam shutters, slits and vacuum systems, were built by Toyama, Japan, and the mirror was supplied by Crystal Scientific, UK. The ACCM system was made by Kohzu, Japan, while the monochromator crystals were supplied by Sarton Works, Japan.

### 2.2. Experimental station   

**Figure 3:**
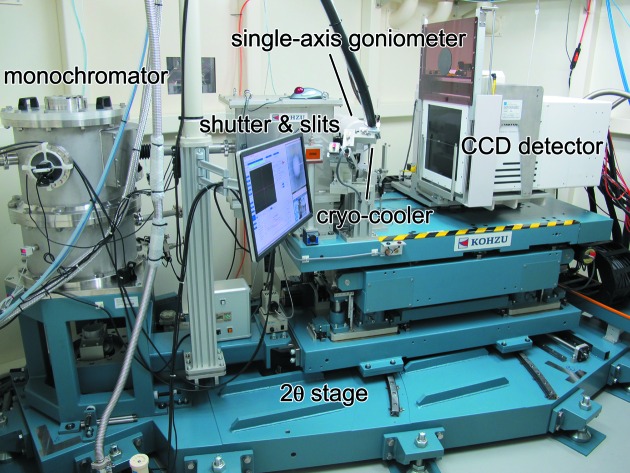
Photograph of the diffractometer of BL2S1, showing the single-axis goniometer, the CCD detector and the cryogenic sample cooler. The monochromator chamber can also be seen to the left.

**Figure 4:**
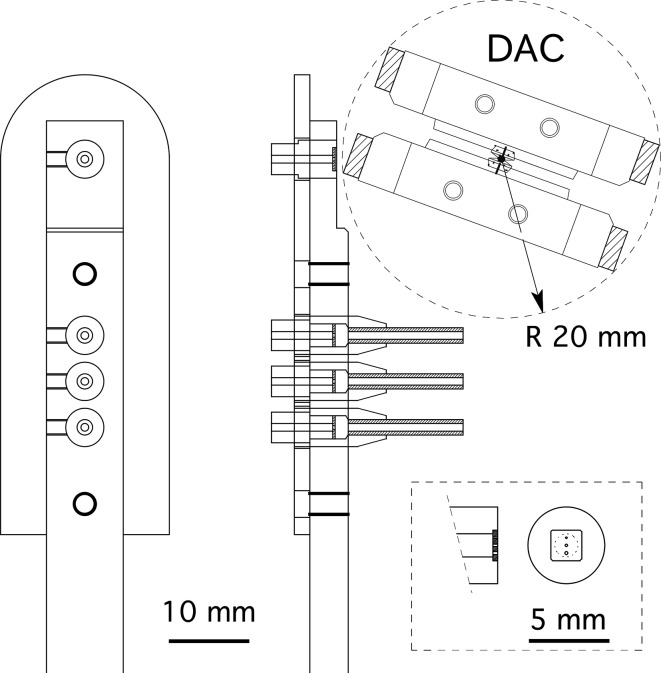
Drawing of the pinhole collimator system of the diffractometer. A magnified view of the set of pinholes without the forward-scatter guard for the DAC experiment is shown in the lower right box.

**Figure 5:**
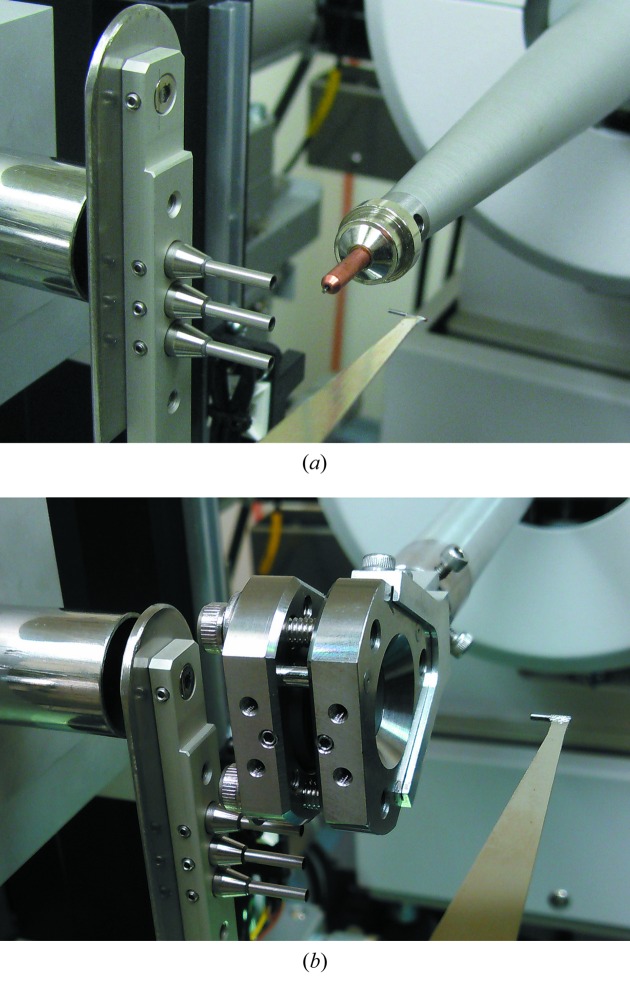
Photographs of the pinhole collimator system of the diffractometer, (*a*) using a standard cryopin for the cryomeasurement and (*b*) using a DAC for the high-pressure experiment.

The diffractometer of BL2S1 is set upon a 2θ stage, which rotates around a center on the θ axis of the ACCM (Fig. 3 ▸). The system consists of a single-axis goniometer (Kohzu, Japan), a cryogenic sample cooler (Rigaku, Japan) and a Quantum 315r CCD detector (ADSC, USA). An indigenously developed pneumatic shutter device using a rotary cylinder is attached to the cryostream nozzle for the flash-cooling and annealing of the sample crystal (Giraud *et al.*, 2009 ▸). The single-axis goniometer consists of a motorized high-precision ω-axis with a resolution of 5 × 10^−5^ degrees per step and a motorized *xyz* stage. The sphere of confusion of the goniometer is less than 5 µm. We do not have a robotic system for crystal mounting/dismounting, but crystal samples on the standard cryopin can be easily mounted on the magnetic head on the rotation axis of the goniometer manually. Using a coaxial CMOS microscope to observe the sample crystal along the X-ray beam, crystal centering with the *xyz* stage of the goniometer is straightforward for general users. As shown in Figs. 4 ▸ and 5 ▸, the collimating system of the diffractometer uses two types of pinhole collimator. One is for standard cryo-measurements and the other is for high-pressure protein crystallography with a diamond anvil cell (DAC) (Girard *et al.*, 2007 ▸). The former consists of three collimators of different pinhole sizes, each with a forward-scatter guard (Fig. 5*a* ▸), designed following the mini-beam collimator developed at beamlines 23-ID-B and D, Advanced Photon Source, USA (Fischetti *et al.*, 2009 ▸). The latter, for the DAC experiment, is just three pinholes in a disk without the forward-scatter guard. This makes it possible to mount a bulky DAC on the goniometer. The standard Merrill–Bassett-type DAC (Merrill & Bassett, 1974 ▸), which has a radius from the center of rotation of less than 20 mm, can be used without interference with the collimator and the beam stop when the beam stop is put 20 mm backward (Figs. 4 ▸ and 5*b* ▸). The absence of a forward-scatter guard does not cause problems. Forward-scattering from the edge of the pinholes can be interrupted by the body of the DAC itself. The diameters of the three pinholes are the same for both collimator types. The three sizes are 0.1, 0.15 and 0.2 mm, and users can choose one of these to best fit their crystal size.

The diffraction data are collected using the Quantum 315r detector mounted on a linear stage allowing sample-to-detector distances between 90 and 342 mm. We also have a pixel array detector, Pilatus 1M (Dectris, Switzerland), but we currently use it as a backup for Quantum 315r. One reason for this is that our chemical crystallography users prefer the larger active area of Quantum 315r. At the detector edge, the maximum 2θ angles accepted by Quantum 315r and Pilatus 1M are 60° and 45°, respectively. The other reason relates to the injection method of the AichiSR. The AichiSR is operated with a top-up injection mode of 300 mA. The focused X-ray beam at the sample position is temporarily lost at the exact moment of injection, since the injection bump covers BL2S1, and it takes about 7 ms to return to the regular orbit. There is no recognizable influence if we use a longer exposure time of 1.0 s per frame or more. However, if we use the Pilatus 1M with shorter exposure time, for example 0.1 s, in order to use its beneficial features, we find that the scaling of the intensity of the diffraction images is noticeably affected by the injection at 1 Hz.

The beamline BL2S1 can also be used for high-pressure protein crystallography. A collection of diffraction images using the DAC can be obtained at shorter wavelengths, usually 0.75 Å with an ACCM of Ge(220). An ancillary pressure measurement device that uses the ruby fluorescence shift (Sigma Koki, Japan) is available beside the experimental hutch.

### 2.3. Control system   

The beamline components and the diffractometer are controlled using the STARS system, which is a client/server beamline control system that operates *via* TCP/IP (Kosuge *et al.*, 2009 ▸). For the remote control of the beamline components such as the VFM, the ACCM and the slits, a STARS client software, *BLC2*, was developed in-house and is used on a Windows PC. A customized version of the user interface, *UGUI*, developed by the macromolecular crystallography group at the Photon Factory (Gaponov *et al.*, 2004 ▸), is used to control the diffractometer and the detectors. This makes it easy for users who have experience using beamlines at the Photon Factory to operate the diffractometer at BL2S1.

The standard data processing softwares used by protein crystallographers, such as *XDS* (Kabsch, 2010 ▸), *iMosflm* (Battye *et al.*, 2011 ▸) and the *CCP4* suite (Winn *et al.*, 2011 ▸), are available on the dedicated Linux desktop PCs at the beamline.

## 3. Conclusions and perspectives   

A protein crystallography beamline has been installed on a superconducting bending-magnet port of the 1.2 GeV AichiSR. The first report on protein crystal structures using this beamline has been published recently (Wachino *et al.*, 2016 ▸). The beamline can be used not only by protein crystallographers but also by chemical crystallographers and other material science communities in the local region. Corporate users may also use the beamline. The beam time application is open two months before the beam time on a first-come first-served basis. At the end of fiscal year 2015, about 80% of the available beam times were provided for user experiments. Of the assigned beam times, about 60% were used by academic and 40% by corporate users. In the era of high-intensity, brilliant micro-beams at third-generation low-emittance synchrotrons, our users can enjoy a modest-size and moderate-intensity beam at BL2S1. Typically, ten datasets can be collected within the operation time of 8.5 h a day; this varies according to the exposure time determined by the crystal diffraction quality.

**Figure 6:**
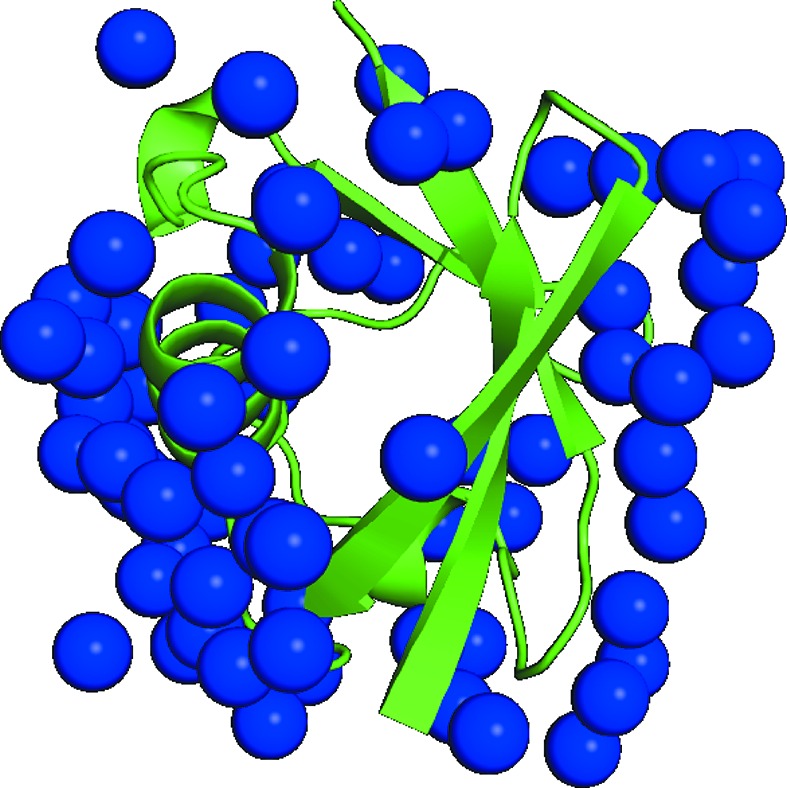
Example of the high-pressure protein crystal structure analyzed using BL2S1. The structure of the ubiquitin molecule at 600 MPa is shown in green. The water molecules are shown in blue.

Authors use these features for the diffraction intensity measurement of radiation-dose-sensitive protein crystals at room temperature. For example, with a combination of the shorter-wavelength X-rays supplied by the Ge(220) monochromator and a relatively larger crystal of several hundred micrometers, we can conduct high-pressure protein crystallography using the DAC without burning the protein crystal with the impact of the intense beams produced at undulator beamlines (Girard *et al.*, 2007 ▸). In Fig. 6 ▸, the crystal structure of human ubiquitin at 600 MPa is shown as an example of our efforts (manuscript in preparation). Data collection parameters and statistics are compiled in Table 3 ▸. A complete high-pressure diffraction dataset can be obtained at BL2S1 with 0.75 Å using the DAC, if crystals of sufficient size are provided.

We do not use a fast detector like the Pilatus 1M with a small-angle oscillation width or the fine slice data collection method, because of the short-time variation of the X-ray beam intensity at injection. The light source division of the Synchrotron Radiation Research Center, Nagoya University, is actively working to make beam injection with a pulsed multipole magnet possible. If top-up injection with the latter method were realised, the beam intensity loss problem caused by the injection could be overcome and the system would become more flexible in its choice of detectors.

## Figures and Tables

**Table 1:** Beamline details

Beamline name	BL2S1
Source type	5 T superconducting bending magnet
Mirror	1.0 m single-crystal Si with Pt coating
Monochromator	Triangular bend asymmetric Ge(111) and Ge(220) single-crystal
Energy range (keV)	7–17
Wavelength range (Å)	0.7–1.8
Beam size (µm)	∼200 × 200 (FWHM, without the pinhole collimator)
Flux (photons s^−1^)†	2.6 × 10^9^ (6.9 keV, 1.80 Å)
	1.1 × 10^10^ (11.1 keV, 1.12 Å)
	4.6 × 10^9^ (16.5 keV, 0.75 Å)
Goniometer	Horizontal single-axis
Cryo capability	Liquid-nitrogen cryogenic sample cooler
Detector	ADSC Quantum 315r and Dectris Pilatus 1M

† After the 200 µm pinhole collimator, and 300 mA top-up operation. It decreases by about 0.7 and 0.4–0.6 times with 150 µm and 100 µm pinholes, respectively.

**Table 2:** Monochromator crystals

Crystal	Asymmetric angle (°)†	Optimum energy / wavelength (keV) / (Å)‡
Ge(111)	7.06	12.7 / 0.98
	7.61	11.8 / 1.05
	13.05	7.0 / 1.78
Ge(220)	8.15	18.0 / 0.69

† The asymmetric angles are the actual values, not the designed ones.

‡ It may be usable but the beam focusing and monochromaticity become incompatible at energy/wavelength deviating from this optimum.

**Table 3:** Data collection statistics of ubiquitin at 600 MPa

Wavelength (Å)	0.75
Temperature (K)	297
Crystal-to-detector distance (mm)	340
Oscillation angle (°)	1.0
Exposure time per image (s)	20
Total rotation range (°)	20
Crystal size used (mm)	0.13 × 0.13 × 0.38

Space group	*P*6_3_22
Cell dimensions (Å)	82.79, 82.79, 55.29
Resolution (Å)	2.00 (2.12–2.00)
Mosaicity (degree)	0.067
*R*_merge_ (%)†	6.7 (31.0)
*I*/σ(*I*)	12.1 (3.3)
Completeness (%)	95.3 (98.6)
Redundancy	2.6

† *R*_merge_ = 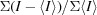.
